# Paralysie complète du nerf oculomoteur révélant un syndrome de Tolosa Hunt

**DOI:** 10.11604/pamj.2015.20.137.6035

**Published:** 2015-02-17

**Authors:** Mariam Anoune, Meriem Abdellaoui, Idriss Andaloussi Benatiya, Hicham Tahri

**Affiliations:** 1 Service d'Ophtalmologie, CHU Hassan II, Fès, Maroc

**Keywords:** Paralysie, oculomotrice, Tolosa-Hunt, palsy, oculomotor, Tolosa-Hunt

## Abstract

Le syndrome de Tolosa Hunt (STH) est une ophtalmoplégie douloureuse due à une inflammation idiopathique et chronique de la fissure orbitaire supérieure ou du sinus caverneux. Le diagnostic répond à des critères cliniques et radiologiques précis. L'habituelle régression spontanée en quelques semaines est spectaculairement raccourcie par la corticothérapie. L'existence même de ce syndrome fut longtemps débattue et son cadre nosologique discuté. Nous rapportons le cas d'une patiente de 65 ans présentant une ophtalmoplégie complète du nerf oculomoteur commun. Le diagnostic de syndrome de Tolosa Hunt a été retenu après avoir éliminé toutes les autres causes de cette ophtalmoplégie, à savoir, les causes tumorales, vasculaires, métaboliques et infectieuses, et aussi devant l'amélioration spectaculaire sous corticothérapie générale. Le syndrome de Tolosa Hunt est maintenant une entité anatomo-clinique bien définie mais très peu fréquente. Il reste un diagnostic d’élimination, il est évoqué après une enquête étiologique minutieuse. Son traitement repose essentiellement sur la corticothérapie générale associé à un suivi méticuleux.

## Introduction

Le syndrome de Tolosa-Hunt (STH) se caractérise par une ophtalmoplégie douloureuse en relation avec un processus inflammatoire non spécifique du sinus caverneux. Ce syndrome est aussi rare que méconnu en milieu ophtalmologique. Il reste un diagnostic d’élimination. Nous rapportons un cas de syndrome de syndrome de Tolosa Hunt à travers lequel nous rappelons les différents aspects diagnostiques, thérapeutiques et évolutifs de cette pathologie.

## Patient et observation

**Figure 1:**
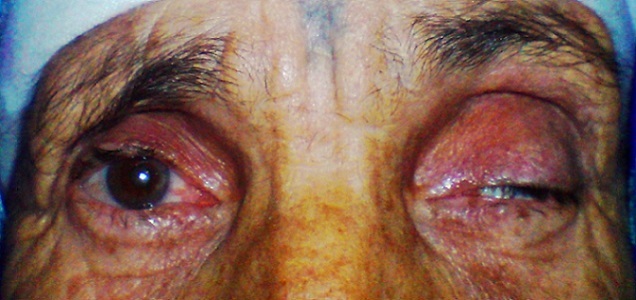
Ptosis gauche

**Figure 2:**
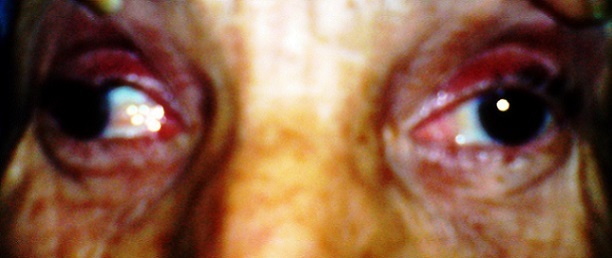
Paralysie du regard en dedans de l’œil gauche

**Figure 3:**
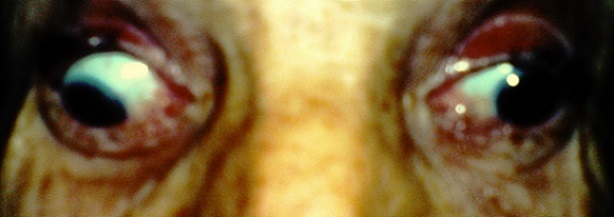
Paralysie du regard en bas de l’œil gauche

**Figure 4:**
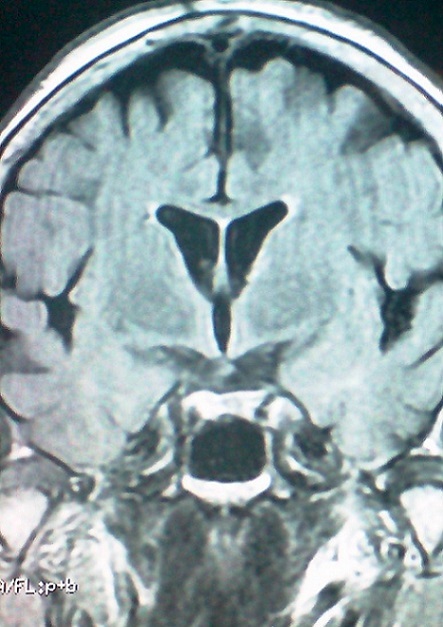
Coupe frontale à l'IRM cérébrale en séquence T1 ne montrant pas d'anomalies au niveau du sinus caverneux

**Figure 5:**
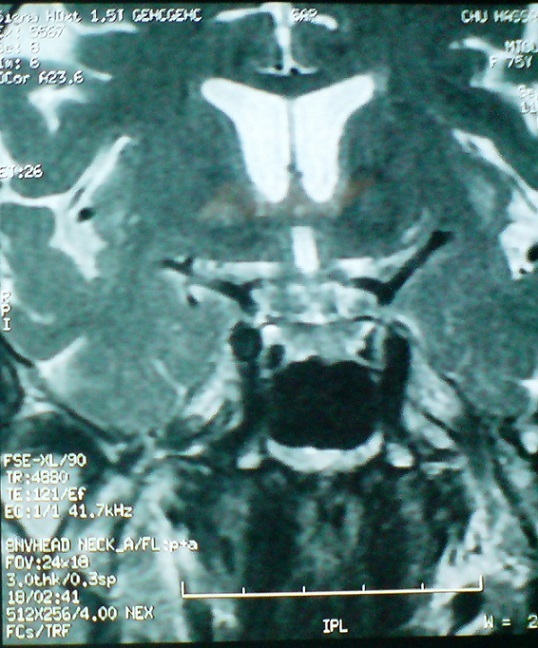
Coupe frontale à l'IRM cérébrale en séquence T2 ne montrant pas d'anomalies au niveau du sinus caverneux

Il s'agit d'une patiente de 65 ans, sans antécédents pathologiques notables, qui a consulte aux urgences ophtalmologiques pour des céphalées unilatérales gauches apparues depuis 5 jours, associées depuis 72 heures à un ptosis gauche (Figure 1). L'examen ophtalmologique a retrouvé une paralysie complète du nerf oculomoteur commun gauche et un ptosis homolatéral avec la mise en évidence d'une diplopie binoculaire lors du soulèvement de la paupière supérieure gauche. On a retrouvé aussi une paralysie du regard vers le haut, en dedans et en bas (Figure 2, Figure 3), l'examen du segment antérieur et du fond d’œil est normal aux deux yeux. L'examen neurologique a objectivé une paralysie des branches 1 et 2 du nerf trijumeau. Le reste de l'examen somatique est sans particularité. La patiente a bénéficié d'une imagerie par résonance magnétique (IRM) cérébrale et une angio-IRM qui sont revenues normales, notamment pas d'atteinte au niveau du sinus caverneux (Figure 4,Figure 5). Une ponction lombaire avec mesure de pression sont sans anomalies. On a complété l'enquête étiologique par un bilan biologique notamment inflammatoire et infectieux (vitesse de sédimentation, Protéine-C-Réactive, numération formule sanguine) ainsi que l'hémoglobine glyquée qui sont normales. Les sérologies virales (notamment herpétiques, cytomégalovirus, VIH) sont négatives. Le bilan immunologique (ANCA, anti DNA natifs), la biopsie des glandes salivaires ainsi que le dosage de l'enzyme de conversion, sont revenus sans particularité. Le diagnostic du syndrome de Tolosa Hunt a été retenu devant la négativité de tous les examens complémentaires réalisés. Notre patiente a été mise sous corticothérapie avec une amélioration de la symptomatologie et une régression spectaculaire des troubles oculomoteurs après 72 heures, sans récidive après 1 an de surveillance.

## Discussion

Le syndrome de Tolosa Hunt est un syndrome rare, son incidence est estimée à un cas par million par an [1]. Il est caractérisé par une ophtalmoplégie douloureuse, due inflammation granulomateuse idiopathique du sinus caverneux. Il y a près de 50 ans, Tolosa [2] a rapporté un cas d'un patient avec des douleurs orbitaires gauches et une ophtalmoplégie totale homolatéral, l'angiographie cérébrale a montré un rétrécissement intra-caverneux de l'artère carotide gauche, après le décès du patient l'autopsie a révélé une inflammation granulomateuse du sinus caverneux. Sept ans plus tard, Hunt et al [3] ont défini une entité clinique d’étiologie obscure suite à l’étude de 6 patients. La réponse thérapeutique aux glucocorticoïdes a été reconnue par Hunt et son équipe. En 1966, Smith et Taxdal [4] ont été les premiers à appliquer l’éponyme « syndrome de Tolosa-Hunt » pour cette entité clinique, sur la base d’étude de 5 cas supplémentaires. Bien que considéré comme une affection bénigne, des déficits neurologiques permanents peuvent se produire, et les rechutes sont possibles, nécessitant ainsi un traitement immunosuppresseur prolongé. Le syndrome de Tolosa-Hunt est causé par un processus inflammatoire d′étiologie inconnue. Sur le plan histologique, il existe une inflammation non spécifique des cloisons et de la paroi du sinus caverneux, avec des granulomes à cellules géantes, et une prolifération des fibroblastes [2, 3]. L′inflammation produit alors une pression et un dysfonctionnement secondaire des structures dans le sinus caverneux, y compris les nerfs crâniens III, IV et VI, ainsi que les divisions supérieures de nerf crânien V. Alors que les rapports d′extension intracrânienne de l′inflammation existent [1], il n′y a pas de rapports d′implication systémique. Des cas de syndrome de Tolosa-Hunt ont été rapportés chez des patients présentant d′autres troubles inflammatoires, telles que le lupus érythémateux disséminé, mais cela peut représenter une simple association de deux maladies auto-immunes [5]. Le STH peut affecter des personnes de tout âge, sans prédominance du sexe. En général il est unilatéral, mais des cas de bilatéralité ont été rapportés [6].

L'International Headache Society décrit l′évolution et les caractéristiques du syndrome de Tolosa-Hunt comme: “douleur orbitale épisodique associée à la paralysie d′un ou plusieurs des troisième, quatrième, et / ou sixième nerfs crâniens qui disparaît généralement spontanément, mais tend à rechute et remettre” [7]. Le diagnostic du STH repose actuellement sur des critères bien précis [6, 8]: La douleur, constante, est unilatérale, orbitaire, non pulsatile, apparaissant en quelques jours; L'ophtalmoplégie associée correspondant à une atteinte: du nerf oculomoteur commun: 80% (comme c'est le cas de notre patiente); du nerf abducens: 70%; du nerf pathétique: 29%; autres nerfs peuvent être touchés: nerf optique, nerfs maxillaire et mandibulaire, nerf facial. L'exclusion d'une autre cause par la neuro-imagerie; L'efficacité spectaculaire de la corticothérapie est évocatrice du diagnostic.

De nombreuses études se sont intéressées au STH et à l'apport de l'imagerie par résonance magnétique, examen indispensable au diagnostic de cette entité, car il permet [9]: d’éliminer d'un processus inflammatoire spécifique (type sarcoïdose) ou tumorale; de montrer l'existence d'une zone d'iso-signal en T1 et T2 et la déformation du sinus caverneux qui sont des signes évocateurs.

Néanmoins, il semble possible que les techniques d'IRM puissent être mises en défaut et ne détectent pas les infiltrats minimes (comme c'est certainement le cas de notre patiente). La non visualisation de ces infiltrats par l'IRM indiquerait un stade précoce de la maladie et donc une meilleure réponse au traitement [10]. La certitude diagnostique peut être donnée par la biopsie qui reste difficile et hasardeuse (mortalité et morbidité élevées) et n'est indiquée qu'en cas de non réponse à une forte dose de corticoïdes. L'efficacité spectaculaire de la corticothérapie est évocatrice mais pas spécifique. D'où la nécessité d'une surveillance prolongée de plusieurs mois pour retenir ce diagnostic de façon définitive. Le STH reste donc un diagnostic d'exclusion, le clinicien doit éliminer les différentes étiologies d'une ophtalmoplégie douloureuse, qui sont multiples, à savoir les causes tumorales (exemple: lymphome), vasculaires, traumatiques, inflammatoires (sarcoïdose, Wegener..), infectieuses et métaboliques [11]. Le traitement consiste en une dose élevée de prednisone par voir orale pendant une durée de 4 semaines. Une amélioration notable est souvent évidente dès les premières 24 heures de traitement, [12] une non réponse aux stéroïdes suggère un autre diagnostic. L'imagerie doit être effectuée tous les 1-2 mois jusqu’à la résolution des anomalies à l'imagerie. Les patients doivent être informés que les rechutes peuvent se produire et à long terme, une nouvelle cure de corticothérapie peut être nécessaire ou parfois même des immunosuppresseurs. Chez notre patiente, après un an de surveillance, on n'a pas constaté de rechute [13].

## Conclusion

Le STH est certainement une entité anatomo-clinique rare, à part, malgré la non connaissance de ses bases étiopathogéniques. L'IRM est un examen précieux grâce à sa meilleure sensibilité pour la détection des lésions du sinus caverneux et sa reproductibilité. Cependant elle demeure non spécifique puisque plusieurs affections peuvent simuler le STH. Le diagnostic de STH est un diagnostic d’élimination avec nécessairement une évaluation précise initiale, un traitement par corticothérapie approprié et un suivi méticuleux.
